# Body Size, Cerebral Blood Flow, Ambient Temperature, and Relative Brain Temperatures in Newborn Infants under Incubator Care

**DOI:** 10.3390/bios14040209

**Published:** 2024-04-22

**Authors:** Satoko Fukaya, Sachiko Iwata, Kennosuke Tsuda, Akiko Hirose, Masahiro Kinoshita, Shinji Saitoh, Osuke Iwata

**Affiliations:** 1 Center for Human Development and Family Science, Department of Pediatrics and Neonatology, Nagoya City University Graduate School of Medical Sciences, Nagoya 467-8601, Japanss11@med.nagoya-cu.ac.jp (S.S.); 2 Centre for Developmental and Cognitive Neuroscience, Department of Paediatrics and Child Health, Kurume University School of Medicine, Kurume 830-0011, Japan

**Keywords:** ambient temperature, body weight, brain temperature, cerebral blood flow, head circumference, near-infrared spectroscopy, preterm infant, rectal temperature, tissue oxygenation index

## Abstract

Subtle changes in body temperature affect the outcomes of ill newborns. However, the temperature profile of neonatal brains remains largely unknown. In open-cot care, increased cerebral perfusion is correlated with higher superficial brain temperatures. This study investigated the dependence of brain temperature (relative to rectal temperature) on ambient temperature, body size, cerebral perfusion, and metabolism in infants receiving incubator care. Rectal, scalp, and brain temperatures, superior vena cava flow, and brain oxygenation were assessed using echocardiography, thermo-compensatory temperature monitoring, and near-infrared spectroscopy in 60 newborns. These infants had a mean postconceptional age of 36.9 (2.2) weeks and weighed 2348 (609) g at the time of evaluation. The ambient temperature was maintained at 30.0 (1.0) °C. A higher rectal temperature was associated with greater postconceptional age (*p* = 0.002), body weight (*p* < 0.001), and head circumference (*p* < 0.001). Relative scalp, superficial brain, and deep brain temperatures were associated with smaller head circumference (*p* < 0.001, *p* = 0.030, and *p* = 0.015, respectively) and superior vena cava flow (*p* = 0.002, *p* = 0.003, and *p* = 0.003, respectively). In infants receiving incubator care, larger head sizes and increased brain perfusion were associated with lower relative scalp and brain temperatures. When considered alongside previous reports, cerebral perfusion may contribute to maintaining stable cerebral tissue temperature against ambient temperature changes.

## 1. Introduction

Newborn infants are susceptible to changes in their body temperature [1,2]. Instances of low body temperature before the commencement of intensive care have been associated with an increased risk of mortality and long-term neurodevelopmental impairment in asphyxiated near-term and term infants [3]. Following the hospitalisation of asphyxiated infants in neonatal intensive care units, a temporary rise in temperature above 38 °C is associated with an increased risk of death or moderate-to-severe neurodevelopmental impairments by 2 years of age [4,5]. Maintaining the body temperature of such infants at 33–34 °C for 72 h increases the likelihood of survival without neurodevelopmental impairments [6]. Precise temperature control is required even after the commencement of cooling, as a 1.0 °C increase in body temperature is associated with an increased odds of 2.1 (95% confidence interval [CI], 1.4–3.2) for death or severe neurodevelopmental impairments by 18 months of age [3]. Thus, accumulating evidence supports the presence of temperature ranges within which the brain is best protected following near-term and term birth asphyxia. A recent study further demonstrated that low admission temperature in very low birth weight infants was associated with mortality or neurodevelopmental impairment by 18 postconceptional months [7]. Because the body temperature of preterm infants is prone to heat loss to their environment, active temperature control using a warm and humid incubator is essential [8]. This type of temperature control, distinct from that required for term infants, is likely to lead to differing spatial temperature distributions within the immature brain. Thus far, few studies have documented the temperature distribution within the immature brain and its relationship with neurodevelopmental outcomes, and therefore, the impact of subtle temperature variations on brain injury in preterm infants can only be inferred from the evidence observed in term asphyxiated infants. However, given the vulnerability of the immature brain to subclinical stressors and the presence of distinct patterns of cerebral injury and neurodevelopmental outcomes in preterm infants [9,10,11], a comprehensive understanding of temperature distribution and its regulation within the immature brain may facilitate the identification of novel mechanisms underlying cerebral injury of prematurity.

The brain temperature is influenced by various factors, including intrinsic heat generation, heat delivery and removal via blood flow, and extrinsic thermal exchange via evaporation, convection, and radiation [12]. A preclinical study investigated the brain temperature distribution in newborn piglets weighing approximately 1.5 kg [13], comparable to that of preterm infants born at approximately 30 weeks of gestation [14]. The study found that smaller piglets had cooler superficial brains, speculating that the brains of smaller animals are more efficiently cooled by ambient air because of higher head surface area-to-volume ratios. However, these piglets were studied under general anaesthesia within a cold magnetic resonance spectrometer bore, rendering their translation into clinical practice for preterm infants difficult. Recent magnetic resonance techniques have enabled 2-dimensional temperature mapping within the neonatal brain [15]. However, infants must be placed within the imaging bore during data acquisition at relatively low ambient temperatures.

Using a thermo-compensatory deep tissue temperature monitor, echocardiography, and near-infrared spectroscopy (NIRS), our research team previously investigated the temperature distributions in the body, scalp, and brain of newborn infants, who were cared for in an open cot with an ambient temperature of 25–26 °C [16]. The study revealed that a higher body weight was associated with a higher rectal temperature. After adjusting local temperature values for rectal temperature, increased cerebral perfusion was associated with a higher superficial brain temperature (relative to rectal temperature), suggesting that blood perfusion is likely to deliver heat to the superficial brain tissue when the ambient temperature is considerably lower than the body temperature. However, given that preterm infants usually receive care in warm, closed incubators during their critical periods [17], understanding the thermal regulation and subsequent brain temperature profiles of incubator-cared infants becomes essential. Investigating thermal regulation within the immature brain under different ambient conditions might uncover novel mechanisms of cerebral injury specific to certain maturational stages and ultimately contribute to developing active brain temperature control strategies to mitigate brain injury in infants.

This study aimed to investigate brain temperature distribution and its relationship with body size, cerebral perfusion, metabolism, and ambient temperature in newborn infants receiving care in closed incubators.

## 2. Materials and Methods

### 2.1. Study Population

Sixty newborn infants without major anomalies or brain lesions were recruited from the special care unit of a tertiary neonatal intensive care centre (Kurume University Hospital, Fukuoka, Japan). The reasons for hospitalisation included low birth weight (n = 36), hypoglycaemia (n = 14), respiratory failure (n = 5), maternal autoimmune disease (n = 3), or feeding problems (n = 2). At the time of the study, all infants were clinically stable and maintained in a closed incubator (Dual Incu I; Atom, Tokyo, Japan).

### 2.2. Data Collection

Data on gestational age, body weight, postnatal age, head circumference at the time of the study, and total blood haemoglobin (Hb) concentration assessed within 3 days of the study were retrieved from electronic patient records. Temperature, echocardiographic, and NIRS data collection followed a previously reported study protocol [16]. Briefly, newborn infants were studied while either asleep or calmly awake, approximately 1 h after feeding. Data were collected by the same research team (echocardiography, S.I. or A.H.; temperature measurement; and NIRS, K.T. or O.I.) in the same order and was completed within 20 min to minimise technical bias.

#### 2.2.1. Temperature Measurements

First, scalp temperature (T_scalp_) was measured at the centre of the forehead using an infrared thermometer (Thermofocus Pro, Technimed, Varese, Italy). Brain temperature was assessed using a zero-heat-flow tissue-core thermometer (Coretemp, Terumo, Tokyo, Japan) with two probes applied to the anterior fontanelle (T_brain-25_, 25 mm in diameter) and forehead (T_brain-15_, 15 mm in diameter). The probe diameter theoretically corresponds to the depth of the tissue, reflecting the temperature [18]. The rectal temperature (T_rectal_) was measured at 3 cm from the anal margin (C202, Terumo, Tokyo, Japan). The ambient temperature was measured in an infant incubator (605-H1 Mini, Testo, Yokohama, Japan).

#### 2.2.2. Echocardiographic Measurements

The blood flow of the superior vena cava (SVC) was measured with an ultrasound system (iE33 and 8–13 MHz vector array transducer, Philips, Amsterdam, The Netherlands) using an established method [19]:

$$SVC\;flow=V_{SVC}\times HR\times \pi \times \frac{\emptyset_{svc}^{2}}{4}$$

where:

*V_svc_* = velocity time integral of SVC in cm,

*HR* = heart rate per minute, and

*Φ*_svc_ = mean SVC diameter in cm.

As a surrogate marker of cerebral blood flow, SVC flow relative to 100 g of brain mass (*rSVC flow*) was calculated using brain weight estimated from the head circumference [20].

#### 2.2.3. NIRS Data Acquisition

Ten-second data acquisition from the forehead was performed in five repetitions, with the probe repositioned each time (mean values used) using a time-resolved NIRS system (TRS-10, Hamamatsu Photonics, Hamamatsu, Japan). The absorption coefficients for the three wavelengths (761, 791, and 836 nm) were calculated to determine the absolute concentrations of the cerebral tissue oxy-, deoxy-, and total Hb. Additionally, the tissue oxygenation index (*TOI*) was computed using the formula:

$$TOI=\frac{\mathrm{oxy}\text{-}\mathrm{Hb}}{\mathrm{oxy}\text{-}\mathrm{Hb}+\mathrm{deoxy}\text{-}\mathrm{Hb}}$$

### 2.3. Data Analysis

Values are presented as the mean (standard deviation) unless specified otherwise. Building on the understanding that core body temperature serves as the primary independent variable of regional brain temperature, and recognising that core body temperature itself is highly influenced by ambient conditions in newborn infants [16], T_brain-25_, T_brain-15_, and T_scalp_ were adjusted for T_rectal_ (T_brain-25_ − T_rectal_, T_brain-15_ − T_rectal_, and T_scalp_ − T_rectal_) to mitigate the impact of core body temperature on peripheral body temperatures. Univariate linear regression analysis was used to assess the dependence of the relative body, scalp, and brain temperatures on postnatal age, postconceptional age, body weight, head circumference, ambient temperature/humidity, blood Hb level, rSVC, and TOI. The statistical results were not corrected for multiple comparisons since the analysis was based on a priori hypotheses and restricted variables. However, findings with *p*-values close to the significance threshold (0.05) were interpreted carefully. To verify the association between cerebral blood flow and relative brain temperature, rather than constructing a prediction model of brain temperature, a multivariable analysis was performed adjusting for predetermined variables of ambient temperature and head circumference as a proxy for brain size.

## 3. Results

Data acquisition was successfully performed on 29 male and 31 female infants. These infants had an average gestational age of 35.6 (3.8) weeks and weighed 2333 (794) g at birth. This study was conducted at 9.2 (14.8) days of life, 36.9 (2.2) weeks postconceptional age, and when the infants weighed 2348 (609) g. However, NIRS data from 11 infants were not used because of technical problems associated with the software.

### 3.1. General Findings

The ambient temperature and humidity within the incubator were 30.0 (1.0) °C and 47.3 (8.2)%, respectively (Table 1). T_rectal_, T_brain-25_, T_brain-15_, T_scalp_, T_brain-25_ − T_rectal_, T_brain-15_ − T_rectal_, and T_scalp_ − T_rectal_ values were 37.0 (0.2), 36.8 (0.2), 36.5 (0.2), 35.5 (0.4), −0.1 (0.2), −0.4 (0.2), and −1.5 (0.4) °C, respectively. The rSVC flow was 85.5 (34.2) mL/100 g/min, and the TOI was 64.9 (3.5)%.

### 3.2. Determinants of T_rectal_

Higher T_rectal_ was associated with female sex (β, −0.137; 95% CI, −0.251 to −0.024), greater postconceptional age (β, 0.040; 95% CI, 0.015 to 0.066), body weight (β, 0.193; 95% CI, 0.108 to 0.278), and head circumference (β, 0.050; 95% CI, 0.028 to 0.072), but not with other variables, including ambient temperature (Table 2).

### 3.3. Determinants of Scalp and Brain Temperatures Adjusted for T_rectal_

**Figure 1:**
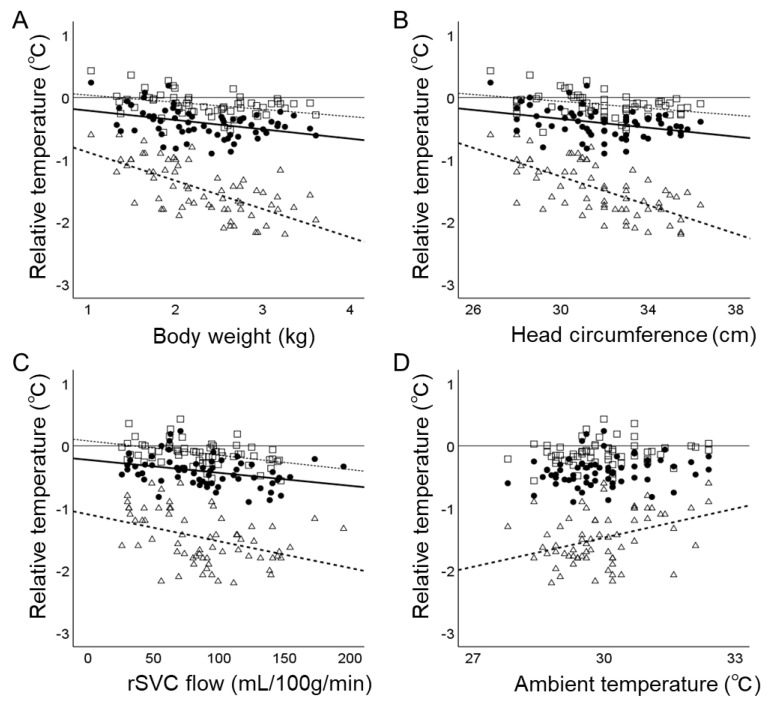
Relationships between relative brain temperature and clinical variables. The relative scalp and brain temperatures to rectal temperature (T_brain-25_ − T_rectal_, T_brain-15_ − T_rectal_, and T_scalp_ − T_rectal_) showed consistent negative relationships with body weight (**A**), head circumference (**B**), and rSVC flow (**C**). T_scalp_ − T_rectal_ showed a positive relationship with the ambient temperature (**D**). Symbols: open triangle, T_scalp_ − T_rectal_; closed circle, T_brain-15_ − T_rectal_; open rectangle, T_brain-25_ − T_rectal_. Regression lines are from the simple linear analysis with *p* < 0.05 for T_scalp_ − T_rectal_ (dashed), T_brain-15_ − T_rectal_ (solid), and T_brain-25_ − T_rectal_ (dotted).

Higher T_brain-25_ − T_rectal_ was associated with smaller body weight (β, −0.115; 95% CI, −0.188 to −0.042), head circumference (β, −0.028; 95% CI, −0.047 to −0.008), ambient humidity (β, −0.006; 95% CI, −0.012 to 0.000), and rSVC flow (β, −0.002; 95% CI, −0.004 to −0.001) (Table 2 and Figure 1).

Higher T_brain-15_ − T_rectal_ was associated with smaller postconceptional age (β, −0.032; 95% CI, −0.058 to −0.006), body weight (β, −0.138; 95% CI, −0.228 to −0.048), head circumference (β, −0.031; 95% CI, −0.055 to −0.008), ambient humidity (β, −0.009; 95% CI, −0.016 to −0.002), rSVC flow (β, −0.003; 95% CI, −0.004 to −0.001), and greater TOI (β, 0.020; 95% CI, 0.005–0.035). Higher T_scalp_ − T_rectal_ was associated with smaller postconceptional age (β, −0.117; 95% CI, −0.156 to −0.079), body weight (β, −0.450; 95% CI, −0.580 to −0.320), head circumference (β, −0.112; 95% CI, −0.147 to −0.078), rSVC flow (β, −0.006; 95% CI, −0.008 to −0.003), higher ambient temperature (β, 0.160; 95% CI, 0.063 to 0.257), and TOI (β, 0.029; 95% CI, 0.084 to 0.186).

In the multivariable analysis, considering the roles of head size and blood flow to the relative scalp and brain temperatures to T_rectal_, smaller head circumference and rSVC flow were found to be associated with higher T_brain-25_ − T_rectal_, T_brain-15_ − T_rectal_, and T_scalp_ − T_rectal_ (Table 2).

## 4. Discussion

In newborn infants receiving care in a closed incubator, we demonstrated that greater body weight, head size, and cerebral blood flow were associated with lower relative scalp and brain temperatures. These relationships were relatively robust for superficial structures. Since greater cerebral blood flow is associated with higher superficial brain temperatures in infants cared for in an open cot [16], cerebral blood flow is likely to deliver heat to brain tissue in cool environments and withdraw heat in warm environments. Further studies are required to address temperature distribution within immature brains associated with physiological and pathological clinical conditions.

### 4.1. Ambient Temperature and Brain Temperature

Proper insulation is essential for newborn infants because of their poor ability to maintain normal body temperatures in cold and dry environments [21,22,23]. Heat exchange actively occurs on the infant’s body surface through evaporation, convection, and radiation [24]. Given the relatively larger surface area of an infant’s head than that of children and adults [25], the fraction of heat exchange occurring at the scalp surface might be large enough to alter the temperature profile within the brain. Indeed, our previous study in 32 infants receiving care in an open cot showed robust relationships between the ambient temperature (25.7 °C on average) and relative scalp and brain temperatures (T_brain-25_ − T_rectal_, T_brain-15_ − T_rectal_, and T_scalp_ − T_rectal_) [16]. However, in our current study, only T_scalp_ − T_rectal_ was dependent on the ambient temperature within the incubator (30.0 °C on average). The ambient conditions within the incubator may have helped minimise heat exchange between the scalp and the environment. Active insulation and high-humidity environments are encouraged to achieve thermo-neutral conditions for the acute phase care of extremely preterm infants [26,27]. However, considering the significant impact that a 1 °C rise in body temperature can have on the outcomes of asphyxiated newborn infants [3,4,5], there might be a threshold brain temperature above which the immature brain becomes susceptible to injury during the critical period. To better understand the benefits and potential adverse effects of current temperature control measures for sick newborns, the establishment of non-invasive techniques enabling continuous monitoring of brain temperature is encouraged.

### 4.2. Body, Head Size, and Brain Temperature

Previous studies have demonstrated positive relationships between body size and core body temperature in preterm and term infants [7,16]. In our study, where the infants were under active temperature control within a closed incubator, the inter-individual differences in core body temperature were expected to be minimal. Indeed, the standard deviations for rectal, brain, and scalp temperatures fell within a narrow range of 0.2 to 0.4 °C. Nonetheless, a robust dependence of T_rectal_ on body size was observed. This association could be attributed to a larger body size correlating with a smaller surface area relative to body mass, leading to reduced heat loss from the surface [12]. Additionally, infants with greater body weight might be more mature and have more adipose tissue, which helps maintain the target body temperature via nonshivering thermogenesis [28,29].

In contrast to the accumulated knowledge on the relationship between body weight and core body temperature [30], limited information is available regarding the relationship between body size and brain temperature in newborn infants. In an experimental study measuring the brain temperature of newborn piglets in a relatively cool environment, greater body weight was associated with higher brain temperatures [13]. However, our previous study on newborn infants did not observe such a dependence of relative brain temperatures on body weight when cared for in an open cot with ambient temperatures of 25–26 °C [16]. In the current study, conducted in a warm incubator, robust relationships were observed between body weight, head circumference, and relative scalp and brain temperatures, with larger body sizes associated with lower relative scalp and brain temperatures. Although statistically insignificant, a trend suggests that the ambient temperature within the incubator was relatively lower for infants with greater body weight. This might imply that smaller infants received relatively higher ambient temperatures, leading to less heat loss on the scalp surface and paradoxically higher relative scalp and brain temperatures. Another explanation is that we analysed the scalp and brain temperatures after adjusting for T_rectal_. Considering the positive relationship between body weight and T_rectal_, using formulas containing T_rectal_ (i.e., T_brain-25_ − T_rectal_, T_brain-15_ − T_rectal_, and T_scalp_ − T_rectal_) might increase the likelihood of establishing relationships between body size and T_rectal_, but not between T_brain-25_, T_brain-15_, and T_scalp_.

### 4.3. Cerebral Blood Flow and Brain Temperature

Our previous study involving hospitalised infants cared for in an open cot demonstrated that greater cerebral blood flow was associated with higher relative brain temperatures, with a more pronounced effect on the superficial structures [16]. In contrast, our current study revealed that greater rSVC flow was associated with lower relative scalp and brain temperatures. At first glance, these findings may appear conflicting. However, since the current study population cared for within a closed incubator exhibits considerably higher ambient temperatures (30.0 °C on average) than those in the previous study (25.7 °C on average) [16], we speculate that cerebral blood flow may play dual roles in heat exchange. Specifically, the cerebral blood flow may deliver heat to warm up the brain in colder ambient temperatures, while it may contribute to cooling the brain in warmer ambient temperatures. Shortly after ejection from the left ventricle, the arterial blood temperature is equivalent to the core body temperature [31], raising uncertainty about whether an increase in cerebral blood flow contributes to tissue cooling. However, studies in human adults have shown that blood temperature is higher in the carotid vein than in the carotid artery [32], suggesting that arterial blood deprives the brain of heat. Zhu and colleagues showed in anaesthetised rats that hypercapnia-induced elevation in cerebral blood flow correlated with increased brain temperature and speculated that a greater cerebral blood flow may contribute to reducing the body core and brain temperature differential [33]. Further studies are needed to delineate the covert roles of cerebral blood flow as a heat buffer for the brain.

### 4.4. TOI and Brain Temperature

The current study observed modest relationships among TOI, T_brain-15_ − T_rectal_, and T_scalp_ − T_rectal_. Theoretically, an increase in TOI is associated with an increase in arterial blood volume and oxygenation and a reduction in venous blood volume and cerebral oxygen consumption [34]. Clinical studies, which measured TOI and cerebral blood flow before and after hypocapnia-induced vasoconstriction, have demonstrated that reduced cerebral arterial blood flow is associated with reduced tissue oxygenation [35]. However, in our current study, higher T_brain-15_ − T_rectal_ and T_scalp_ − T_rectal_ were simultaneously associated with lower rSVC flow and paradoxically higher TOI of the brain. Donadello and colleagues investigated the relationship between brain perfusion and metabolism in anaesthetised young swine and found that induced hypothermia was associated with reduced cerebral blood flow and increased tissue oxygenation [36]. As discussed in the preceding paragraph, increased cerebral blood flow may potentially decrease superficial brain temperature in a warm environment. Nonetheless, this scenario is more likely to result in diminished cerebral oxygen consumption and, consequently, increased TOI. Currently, we do not have sufficient knowledge or data to explain the mechanism underlying the simultaneous increases in brain temperature and TOI. Considering the relatively less robust relationships observed between scalp and brain temperatures and TOI, our findings should be reassessed in future studies. To address this question, we are conducting a clinical study to investigate the direct relationships among cerebral temperature profiles, cerebral blood flow, and brain oxygen metabolism before and after changes in environmental conditions.

### 4.5. Limitations

This study had a few limitations that should be considered when interpreting the findings. First, because of ethical concerns, patients were recruited following stabilisation and successful weaning from mechanical ventilation. This process may introduce a selection bias towards a greater postnatal age in relatively more immature infants. Additionally, we could not collect data during the critical period when immature brains were most susceptible to injury. Second, repeated data collection from the same patient was impossible for technical reasons. Considering that the core body temperature of hospitalised newborn infants is relatively uniform due to active temperature control within closed incubators, incorporating both inter- and intra-individual differences in the variables may be advantageous for enhancing statistical power to identify subtle changes in the relationship between regional temperatures and other clinical variables. Third, we could not collect NIRS data for 11 newborns, potentially reducing the analytical power of our study. Fourth, akin to most other physiological variables, tissue perfusion and oxygenation fluctuate within a spectrum of frequencies [37]. Although we engineered the data acquisition process to attenuate temporal oscillations of these variables, we were unable to account for the influence of oscillations with cycles of longer than hours [38]. Finally, we used zero-heat-flux core temperature monitoring as a surrogate for deep brain temperature. Although the accuracy of this technique has been validated in various settings [18,39,40,41,42], further studies are required to confirm our findings using new non-invasive techniques, such as magnetic resonance spectroscopy.

## 5. Conclusions

In a cohort of hospitalised newborns cared for in a closed incubator with relatively higher ambient temperatures of approximately 30 °C, our study revealed that greater body weight, head circumference, and cerebral blood flow were associated with lower relative scalp and brain temperatures. When considered alongside previous findings involving newborns cared for with an ambient temperature of 25–26 °C, where greater cerebral perfusion was associated with higher relative superficial brain temperatures [16], our findings suggest that cerebral blood flow may serve as a heat buffer. Specifically, cerebral blood flow may either help warm the brain tissue in a cool environment or cool the brain tissue in a warm environment. Further studies should address the precise temperature distribution within immature brains. Additionally, they should elucidate whether specific temperature profiles are associated with pathological conditions and adverse outcomes attributed to specific maturation stages.

## Figures and Tables

**Table 1:** Clinical backgrounds and physiological variables.

	Variables		Mean	Standard Deviation
Clinical variables at birth				
	Gestational age (week)		35.6	3.8
	Body weight (g)		2333	794
	Apgar scores	1 min	7.5	1.6
		5 min	8.6	1.1
Clinical variables at the time of the study				
	Postnatal age (d)		9.2	14.8
	Postconceptional age (week)		36.9	2.2
	Body weight (g)		2348	609
	Head circumference (cm)		31.9	2.3
Incubator environment				
	Ambient temperature (°C)		30.0	1.0
	Ambient humidity (%)		47.3	8.2
Temperature measures (°C)				
	T_rectal_		37.0	0.2
	T_brain-25_		36.8	0.2
	T_brain-15_		36.5	0.2
	T_scalp_		35.5	0.4
	T_brain25_ − T_rectal_		−0.1	0.2
	T_brain15_ − T_rectal_		−0.4	0.2
	T_scalp_ − T_rectal_		−1.5	0.4
Haemoglobin, blood flow, and tissue oxygenation				
	Blood Haemoglobin (g/dL)		17.2	2.4
	rSVC flow (mL/100 g/min)		85.5	34.2
	Tissue oxygenation index (%)		64.9	3.5

Abbreviations: T_rectal,_ rectal temperatures. T_brain-25_ and T_brain-15_, brain temperatures, measured using a zero-heat flux thermometer with probe diameters of 25 mm and 15 mm. T_scalp_, scalp temperatures at the forehead. rSVC flow, the relative blood flow of the superior vena cava.

**Table 2:** Dependence of relative body, scalp, and brain temperatures on clinical variables. Abbreviations: T_rectal,_ rectal temperature. T_brain-25_ and T_brain-15_, brain temperatures, measured using a zero-heat flux thermometer with probe diameters of 25 mm and 15 mm. T_scalp_, scalp temperatures at the forehead. rSVC flow, the relative blood flow of the superior vena cava.

Variables		T_rectal_		T_brain25_ − T_rectal_		T_brain15_ − T_rectal_		T_scalp_ − T_rectal_	
		Coefficient × 10^2^	*p*	Coefficient × 10^2^	*p*	Coefficient × 10^2^	*p*	Coefficient × 10^2^	*p*
**Univariable analysis**									
Clinical variables at the time of the study									
	Female sex	−13.7	0.017	1.4	0.778	−1.7	0.777	8.8	0.406
		(−25.1 to −2.4)		(−8.2 to 10.9)		(−13.4 to 10.0)		(−12.0 to 29.6)	
	Postnatal age (d)	−0.3	0.113	0.0	0.771	0.3	0.151	0.6	0.087
		(−0.7 to 0.1)		(−0.3 to 0.4)		(−0.1 to 0.7)		(−0.1 to 1.3)	
	Postconceptional age (week)	4.0	0.002	−1.6	0.141	−3.2	0.016	−11.7	<0.001
		(1.5 to 6.6)		(−3.8 to 0.5)		(−5.8 to −0.6)		(−15.6 to −7.9)	
	Body weight (kg)	19.3	<0.001	−11.5	0.002	−13.8	0.003	−45.0	<0.001
		(10.8 to 27.8)		(−18.8 to −4.2)		(−22.8 to −4.8)		(−58.0 to −32.0)	
	Head circumference (cm)	5.0	<0.001	−2.8	0.005	−3.1	0.010	−11.2	<0.001
		(2.8 to 7.2)		(−4.7 to −0.8)		(−5.5 to −0.8)		(−14.7 to −7.8)	
Incubator environment									
	Ambient temperature (°C)	1.0	0.735	3.4	0.153	4.2	0.152	16.0	0.001
		(−4.9 to 7.0)		(−1.3 to 8.2)		(−1.6 to 10.1)		(6.3 to 25.7)	
	Ambient humidity (%)	0.6	0.115	−0.6	0.037	−0.9	0.012	−1.2	0.051
		(−0.1 to 1.3)		(−1.2 to 0.0)		(−1.6 to −0.2)		(−2.5 to 0.0)	
Haemoglobin, blood flow, and tissue oxygenation									
	Blood haemoglobin (g/dL)	1.5	0.264	0.5	0.623	−1.4	0.244	−3.8	0.084
		(−1.1 to 4.1)		(−1.4 to 2.4)		(−3.8 to 1.0)		(−8.0 to 0.5)	
	rSVC (mL/100 g/min)	0.1	0.244	−0.2	<0.001	−0.3	<0.001	−0.6	<0.001
		(−0.1 to 0.3)		(−0.4 to −0.1)		(−0.4 to −0.1)		(−0.8 to −0.3)	
	Tissue oxygenation index (%)	−0.1	0.949	0.7	0.315	2.0	0.008	2.9	0.040
		(−1.8 to 1.7)		(−0.6 to 1.9)		(0.5 to 3.5)		(8.4 to 18.6)	
**Multivariable analysis**									
	Head circumference (cm)	5.0	<0.001	−2.3	0.015	−2.5	0.030	−9.8	<0.001
		(2.8 to 7.2)		(−4.1 to −0.4)		(−4.8 to −0.2)		(−12.9 to −6.8)	
	Ambient temperature (°C)	4.6	0.119	−0.8	0.742	−0.8	0.781	6.4	0.116
		(−1.2 to 10.3)		(−5.5 to 3.9)		(−6.7 to 5.0)		(−1.6 to 14.3)	
	rSVC (mL/100 g/min)	0.1	0.240	−0.2	0.003	−0.3	0.003	−0.4	0.002
		(−0.1 to 0.3)		(−0.4 to −0.1)		(−0.4 to −0.1)		(−0.6 to −0.1)	

## Data Availability

The data presented in this study are available upon request from the corresponding author.
